# Surgical and Pre-surgical Factors Affecting Appendectomy Outcomes in Jeddah, Saudi Arabia: A Retrospective Record Review

**DOI:** 10.7759/cureus.62960

**Published:** 2024-06-23

**Authors:** Hussain A Alkhalifah, Khalid M Aljehani, Sultan R Algethami, Saud A Alyahya, Abdulaziz A Alzubide, Rayan M Alharbi, Hassan A Khafaji, Fatma K Althoubaity

**Affiliations:** 1 Faculty of Medicine, King Abdulaziz University, Jeddah, SAU; 2 Department of Surgery, King Abdulaziz University Hospital, Jeddah, SAU

**Keywords:** saudi arabia, surgery, histopathology, laparoscopic, appendectomy

## Abstract

Background: Appendectomy is the preferred treatment for acute uncomplicated appendicitis and the most common emergency abdominal surgery. While previous studies have investigated variables affecting post-appendectomy complications, local research is limited, and data on complication rates are scarce. Therefore, this study aimed to investigate appendectomy outcomes and the factors influencing them.

Methods: This retrospective record review included all patients who underwent appendectomies at our center between 2013 and 2023, excluding those who underwent appendectomies as part of other procedures. Data were retrieved from the hospital database and recorded on predesigned Google Forms.

Results: A total of 556 patients were included. Complications occurred in 60 patients (10.8%); the most common included intra-abdominal collections (n=19, 3.4%), postoperative fever (n=13, 2.3%), and surgical site infections (n=11, 2.0%). The most frequently documented histopathological diagnoses included acute appendicitis (n=402, 72.3%), perforated appendicitis (n=109, 19.6%), and gangrenous appendicitis (n=19, 4.4%). Surgical site infection rates were higher after open appendectomies (6.0% vs. 0.9%, P=0.006), while intra-abdominal collections were more frequent after laparoscopic appendectomies (4.1% vs. 0.0%, P=0.095). Additionally, histopathology results showing complicated or chronic appendicitis were associated with higher complication rates (P<0.001, odds ratio=3.793, 95% confidence interval=1.957-7.350).

Conclusion: To the best of our knowledge, this is the largest retrospective review of appendectomy cases in Saudi Arabia. However, this study was conducted in a tertiary care center, which may have caused the rates of complications to appear lower than those in primary centers. We recommend a multi-center study be conducted to establish more accurate results.

## Introduction

Appendectomy is the most frequently performed emergency abdominal surgery [1,2]. The lifetime risk of developing acute appendicitis is 8.6% for males and 6.7% for females, with appendectomy rates of 12% and 23.1%, respectively [3]. Acute appendicitis, characterized by vermiform appendix inflammation, is a surgical emergency requiring the removal of the appendix via laparotomy or laparoscopy. The mortality rate for untreated patients is high, primarily due to ruptures leading to peritonitis and shock [4,5].

Appendectomy remains the preferred treatment for acute uncomplicated appendicitis, with a low complication rate and relatively short hospital stay [6,7]. Some studies have compared different approaches to appendectomies and their outcomes. A 2010 study showed a lower wound infection rate in laparoscopic appendectomy (0%) than in open appendectomy (12.9%) [8]. Additionally, variations in appendix removal procedures may lead to different outcomes, with other factors and variables influencing surgical results. A 2003 Saudi Arabian study found equal intra-abdominal infection rates in laparoscopic and open appendectomy [9].

Some studies have explored factors affecting appendectomy outcomes. Alnajashi et al., in a 2020 study in Riyadh, Saudi Arabia, examined the role of sex, age, operative date, body mass index (BMI), and temperature upon admission on appendectomy complications. Their results showed a significant relationship between BMIs and surgery durations, potentially leading to more complications [6]. Azodi et al., in a 2008 Swedish study, assessed for complications following open appendectomies in patients with increased BMIs and a history of smoking. Smokers and obese patients showed higher complication rates (7.3% and 9.8%, respectively) compared to nonsmokers and average-weight patients (3.8% and 3.6%, respectively) [10].

Previous literature established a link between epidemiological factors, pre-surgical variables, and surgical variables on appendectomy outcomes. However, earlier studies considered a limited number of complications and involved limited variables, and most studies in Saudi Arabia had relatively small sample sizes. As such, the current study aimed to (1) determine the impact of histopathological reports on appendectomy outcomes, and (2) assess the effects of sex, age, nationality, BMI, temperature upon admission, duration of surgery, chronic illnesses, and type of surgery (open vs. laparoscopic) on complications following appendectomies.

## Materials and methods

Study design, setting, and population

This retrospective record review included all patients who underwent appendectomies between January 2013 and January 2023 at our institution in Jeddah, Saudi Arabia. Patients who underwent appendectomies as part of another procedure were excluded. The medical records of all eligible patients were reviewed.

Of the 733 patients who underwent appendectomies during the study period, 121 were excluded due to missing preoperative or postoperative notes in their files, and one was excluded due to an invalid file number. After applying the exclusion criteria, 55 more patients were excluded because their appendectomies were performed as part of another procedure. Ultimately, 556 patients were included in this study.

Data collection method

Patient data were collected from the hospital’s database. Predesigned Google Forms were prepared to manage the following data: (1) demographic and clinical characteristics, including age at the time of surgery, sex, nationality (Saudi or non-Saudi), BMI, and presence of comorbidities; (2) preoperative data including date and time of admission, Caprini score, temperature upon admission, hemoglobin level (Hb), creatinine level, white blood cell (WBC) count, C-reactive protein (CRP) level, and whether preoperative venous thromboembolism (VTE) prophylaxis and prophylactic antibiotics were administered; (3) operative data including date and time of surgery, type of surgery (open, laparoscopic, or other), and duration of surgery (minutes); and (4) postoperative data including histopathology report, whether postoperative VTE prophylaxis and prophylactic antibiotics were administered, and whether any complications occurred.

Patients were followed up for 30 days after surgery to assess complications, classified according to the Clavien-Dindo classification [11].

Data analysis and entry

Data was entered using Microsoft Excel 2021 (Microsoft Corporation, Redmond, WA, USA). IBM SPSS Statistics version 27 (IBM Corp., Armonk, NY, USA) was used for coding and data analysis. Categorical data, such as sex, nationality, comorbidities, pre-and postoperative VTE prophylaxis and prophylactic antibiotics, type of surgery, and histopathology reports, are expressed as frequencies. Continuous data, including age, BMI, temperature, and laboratory results, are defined as means and standard deviations (SDs) for normally distributed values or as medians and interquartile ranges (IQRs) for values that were not normally distributed. Bivariate analysis was conducted for categorical variables using the chi-squared test to identify the associations between categorical variables, including the type of surgery and histopathology findings, and the complication rates. An independent t-test was performed to identify the effect of normally distributed continuous variables, such as Hb level and WBC count, on the complication rate, while the Mann-Whitney U test was used for non-normally distributed continuous variables such as age, BMI, temperature, and the rest of the laboratory results. Multivariate binary logistic regression was used to adjust the P-values and determine the factors independently associated with higher odds of developing complications. Statistical significance was set at P<0.05.

Research ethics

This study was approved by the Institutional Review Board of our facility (no. 336-23). Data remained confidential and were accessible only to the named authors. Privacy and confidentiality were maintained throughout the study, and the data were only accessed by the authors. The requirement for informed consent was waived due to the retrospective nature of the study. This study was conducted in accordance with the ethical principles mentioned in the Declaration of Helsinki (2013).

## Results

Patient characteristics

This study included 556 patients who underwent appendectomies; 340 were males (60.1%) and 222 were females (39.9%). The median age at the time of surgery was 21.00 (14.00-31.00) years, with the youngest and oldest at two and 85 years of age, respectively. Additionally, 54 patients had comorbidities (9.7%). Table 1 summarizes the patients' sociodemographic and clinical characteristics.

**Table 1:** Patient demographics and clinical characteristics. Notes: Age and body mass index data are expressed as median and interquartile range (IQR), and others are expressed as numbers (N) percentages (%). Abbreviations: N: number, IQR: interquartile range.

Variables	Median	IQR
Age at the time of surgery (years)	21.00	14.00–31.00
Body mass index (kg/m^2^)	24.22	20.70–27.55
	N	%
Sex		
Male	334	60.1%
Female	222	39.9%
Age group (years)		
2–14	151	27.2%
15–25	203	36.5%
26–35	111	20.0%
36–45	48	8.6%
46–55	22	4.0%
56 and above	21	3.8%
Nationality		
Saudi	384	69.1%
Non-Saudi	172	30.9%
Past abdominal surgery		
Yes	38	6.8%
No	518	93.2%
Comorbidities		
Hypertension	25	4.5%
Ischemic heart disease	12	2.2%
Diabetes mellitus	22	4.0%
Hyperlipidemia	5	0.9%
Irritable bowel syndrome	3	0.5%
Ulcerative colitis	1	0.1%
Asthma	3	0.5%
Other	14	2.5%

Surgical, pre-surgical, and post-surgical factors and outcomes

Laparoscopic appendectomy was the most common procedure, performed in 463 patients (83.3%). In comparison, 83 patients (14.9%) underwent open appendectomies, five patients (0.9%) underwent exploratory laparotomies, and five patients (0.9%) underwent laparoscopic appendectomies converted to open appendectomies. The operation duration ranged from 26 to 343 minutes, with a median time of 90.00 (72.00-114.00) minutes. On admission, the median temperature was 36.90 (36.60-37.00)°C, the mean WBC count was 13.47±4.92 K/uL, and the median CRP level was 57.60 (10.74-143.50) mg/L. Most patients received preoperative (n=465, 83.6%) and postoperative (n=483, 86.9%) prophylactic antibiotics. However, a few patients received preoperative (n=60, 10.8%) or postoperative (n=208, 37.4%) VTE prophylaxis.

**Figure 1:**
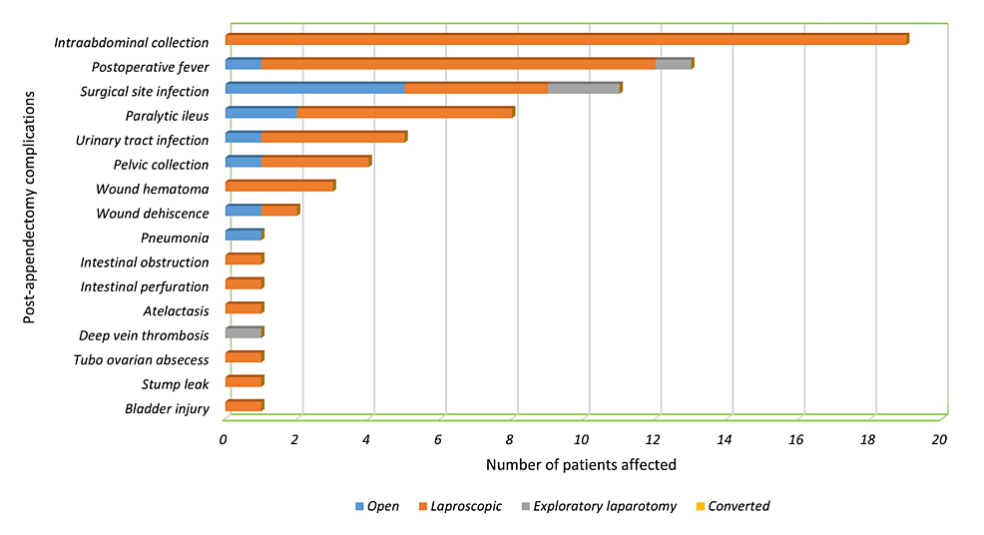
Prevalence of post-appendectomy complications.

Complications occurred in 60 patients (10.8%), with only a few experiencing multiple complications simultaneously. The most common complications were intra-abdominal collections (n=19, 3.4%), postoperative fever (n=13, 2.3%), and surgical site infections (SSIs) (n=11, 2.0%). Additionally, one patient had an iatrogenic bladder injury. Clavien-Dindo grade I accounted for 31.7% of complications, grade II for 26.7%, grade III for 38.3%, and grade IV for 3.3%. Figure 1 illustrates the prevalence of appendectomy complications between the different types of surgeries.

Histopathology reports showed that the diagnoses included acute appendicitis (n=402, 72.3%), perforated appendicitis (n=109, 19.6%), gangrenous appendicitis (n=19, 4.4%), normal appendices (n=16, 2.9%), necrotizing appendicitis (n=5, 0.9%), chronic appendicitis (n=4, 0.7%), and one case (0.2%) of low-grade appendiceal mucinous neoplasm (LAMN). Table 2 summarizes the surgical, pre-surgical, and post-surgical features.

**Table 2:** Surgical, pre-surgical, and post-surgical characteristics. Notes: Continuous data are expressed as mean±standard deviation (SD) or median and interquartile range (IQR), and others are expressed as numbers (N) percentages (%). Abbreviations: CRP: C-reactive protein, Hb: hemoglobin, N: number, SD: standard deviation, IQR: interquartile range, VTE: venous thromboembolism, WBC: white blood cell count.

Variables	Median	IQR
Temperature (°C)	36.90	36.60–37.00
Creatinine (umol/L)	62.00	46.00–78.00
CRP (mg/L)	57.60	10.74–143.50
Duration of surgery (min)	90.00	72.00–114.00
Hospital stay (days)	3.00	2.00–4.00
	Mean	SD
Hb (g/dL)	13.28	1.800
WBC (K/uL)	13.47	4.921
	N	%
Caprini score		
Low	228	94.2%
Moderate	12	5.0%
High	2	0.8%
Preoperative antibiotics		
Yes	465	83.6%
No	91	16.4%
Postoperative antibiotics		
Yes	483	86.9%
No	73	13.1%
Preoperative VTE prophylaxis		
Yes	60	10.8%
No	496	89.2%
Postoperative VTE prophylaxis		
Yes	208	37.4%
No	348	62.6%
Day of surgery		
Weekday	410	73.7%
Weekend	146	26.3%
Type of surgery		
Open appendectomy	83	14.9%
Laparoscopic appendectomy	463	83.3%
Laparoscopic appendectomy converted into open	5	0.9%
Exploratory laparotomy	5	0.9%
Histopathology		
Acute appendicitis	402	72.3%
Perforated appendicitis	109	19.6%
Gangrenous appendicitis	19	3.4%
Necrotising appendicitis	5	0.9%
Chronic appendicitis	4	0.7%
Normal appendix	16	2.9%
Neoplasm	1	0.2%
Complicated patient		
Yes	60	10.8%
No	496	89.2%
Clavien–Dindo		
Grade I	19	31.7%
Grade II	16	26.7%
Grade III	23	38.3%
Grade IV	2	3.3%

Factors influencing complications following appendectomy

Surgery type significantly influenced the overall complication rate, with patients who underwent exploratory laparotomies having a 60% complication rate, compared to 12.0% for open appendectomies, 10.2% for laparoscopic appendectomies, and 0.0% for converted operations (P=0.029). However, when comparing the complication rates of open versus laparoscopic appendectomies, open appendectomies had an insignificantly slightly higher rate of complications (12.0% vs. 10.2%, P=0.745). However, patients who underwent open appendectomies had significantly higher SSI rates than those who underwent laparoscopic surgeries (6.0% vs. 0.9%, P=0.006). In contrast, intra-abdominal collections were higher after laparoscopic than open appendectomies, but the difference was not statistically significant (4.1% vs. 0.0%, P=0.095).

Additionally, the duration of the operation significantly affected the incidence of complications, occurring more frequently in patients with longer operation durations (105.50 (80.50-144.00) minutes vs. 88.50 (71.00-111.00) minutes, P=0.001). Moreover, higher WBC count and CRP levels on admission resulted in higher complication rates (WBC counts: 15.58±6.48 K/uL vs. 13.21±1.80 K/uL, P=0.008; CRP levels: 147.00 (89.27-235.25) mg/L vs. 39.30 (8.77-123.00) mg/L, P=0.001). Complication rates were notably higher in two distinct age groups: individuals aged 2-14 years (pediatrics) and those aged 56 years or older, considered as the extreme age ranges in this analysis. The complication rates for these groups were 15.9% and 14.3%, respectively, compared to 8.6% in the rest (P=0.036).

Histopathology of the appendix indicated a significant effect on the complication rate, as pathologies including perforations, gangrene, and necrosis were associated with higher rates of complications than uncomplicated acute appendicitis (26.6%, 21.1%, 20.0% vs. 6.0%, P<0.001). Furthermore, complications resulted in significantly longer hospital stays, with patients experiencing complications staying in the hospital for a median of 7.00 (3.25-14.00) days, compared with 2.00 (2.00-4.00) days in those without complications (P<0.001). Table 3 demonstrates the relationship between the study variables and the occurrence of post-appendectomy complications.

**Table 3:** Effect of different variables on the occurrence of post-appendectomy complications. Notes: Continuous data are expressed as mean±standard deviation (SD) or median and interquartile range (IQR), and others are expressed as numbers (N) percentages (%). Abbreviations: BMI: body mass index, CRP: C-reactive protein, Hb: hemoglobin, N: number, %: percentage, SD: standard deviation, IQR: interquartile range, VTE: venous thromboembolism, WBC: white blood cell count *Statistical significance was set at p<0.05.

Variables	Complicated (median (IQR))	Not complicated (median (IQR))	P-value
Age at the time of surgery (years)	17.00 (10.25–31.75)	21.00 (14.00–31.00)	0.038*
BMI (kg/m^2^)	23.44 (17.93–27.34)	24.22 (20.76–27.68)	0.081
Temperature (°C)	36.90 (36.52–37.15)	36.80 (36.60–37.00)	0.600
Creatinine (µmol/L)	58.00 (42.00–75.00)	62.00 (47.00–78.30)	0.149
CRP (mg/L)	147.00 (89.27–235.25)	39.30 (8.77–123.00)	0.001*
Duration of surgery (min)	105.50 (80.50–144.00)	88.50 (71.00–111.00)	0.001*
Hospital stay (days)	7.00 (3.25–14.00)	2.00 (2.00–4.00)	<0.001*
	Complicated (mean±SD)	Not complicated (mean±SD)	P-value
Hb (g/dL)	12.85±1.754	13.33±1.800	0.051
WBC (K/uL)	15.58±6.481	13.21±4.641	0.008*
	Complicated (N (%))	Not complicated (N (%))	P-value
Sex			
Male	34 (10.2%)	300 (89.8%)	0.667
Female	26 (11.7%)	196 (88.3%)	
Age group (years)			
2-14	24 (15.9%)	127 (84.1%)	0.036*
15-55	33 (8.6%)	351 (91.4%	
56 and older	3 (14.3%)	18 (85.7%)	
Nationality			
Saudi	34 (8.9%)	350 (91.1%)	0.040*
Non-Saudi	26 (15.1%)	146 (84.9%)	
Past abdominal surgery			
Yes	4 (10.5%)	34 (89.5%)	1.000
No	56 (10.8%)	462 (89.2%)	
Presence of comorbidities			
Yes	7 (13.0%)	47 (87.0%)	0.756
No	53 (10.6%)	449 (89.4%)	
Caprini score			
Low	17 (7.5%)	211 (92.5%)	0.100
Moderate	3 (25.0%)	9 (75.0%)	
High	0 (0.0%)	2 (100%)	
Preoperative antibiotics			
Yes	50 (10.8%)	415 (89.2%)	1.000
No	10 (11.0%)	81 (89.0%)	
Postoperative antibiotics			
Yes	52 (10.8%)	431 (89.2%)	1.000
No	8 (11.0%)	65 (89.0%)	
Preoperative VTE prophylaxis			
Yes	8 (13.3%)	52 (86.7%)	0.652
No	52 (10.5%)	444 (89.5%)	
Postoperative VTE prophylaxis			
Yes	21 (10.1%)	187 (89.9%)	0.789
No	39 (11.2%)	309 (88.8%)	
Day of surgery			
Weekday	47 (11.5%)	363 (88.5%)	0.484
Weekend	13 (8.9%)	133 (91.1%)	
Type of surgery			
Open appendectomy	10 (12.0%)	73 (88.0%)	0.029*
laparoscopic appendectomy	47 (10.2%)	416 (89.8%)	
Laparoscopic appendectomy converted into open appendectomy	0 (0.0%)	5 (100%)	
Exploratory laparotomy	3 (60.0%)	2 (40.0%)	
Histopathology			
Acute appendicitis	24 (6.0%)	378 (94.0%)	<0.001*
Perforated appendicitis	29 (26.6%)	80 (73.4%)	
Gangrenous appendicitis	4 (21.1%)	15 (78.9%)	
Necrotising appendicitis	1 (20.0%)	4 (80.0%)	
Chronic appendicitis	0 (0.0%)	4 (100%)	
Normal appendix	2 (12.5%)	14 (87.5%)	
Neoplasm	0 (0.0%)	1 (100%)	

Multivariate regression analysis was performed to determine independent variables associated with higher complication rates. The model included sex, age, BMI, nationality, temperature upon admission, preoperative Hb level, preoperative WBC count, preoperative creatinine level, type of surgery (laparoscopic vs. open), duration of surgery (minutes), and histopathology of the appendix (simple acute or normal vs. complicated or chronic). The analysis showed that even after regression analysis, a longer duration of surgery (P=0.016, odds ratio (OR)=1.010, 95% confidence interval (CI)=1.002-1.018) and histopathology results indicating complicated or chronic appendicitis (P=<0.001, OR=3.793, 95% CI=1.957-7.350) were both still associated with higher complication rates post-appendectomy. However, after regression analysis, differences in nationality (P=0.188, OR=0.646, 95% CI=0.338-1.238) and WBC counts (P=0.080, OR=1.010, 95% CI=0.994-1.120) showed no significant effect on complication rates. Moreover, the type of surgery did not influence the complication rates (P=0.711, OR=1.175, 95% CI=0.500-2.762). Table 4 summarizes the results of the multivariate analysis.

**Table 4:** Multivariate analysis of variables influencing the rate of complications. Abbreviations: BMI: body mass index, Hb: hemoglobin, WBC: white blood cells, OR: odds ratio, CI: confidence interval, Ref: reference. *Statistical significance was set at p<0.05.

Variables	Occurrence of complications		
	OR	95% CI	P-value
Sex			
Male	1.099	0.505–2.389	0.813
Female	Ref	Ref	Ref
Nationality			
Saudi	0.646	0.338–1.238	0.188
Non-Saudi	Ref	Ref	Ref
Type of surgery			
Open	1.175	0.500–2.762	0.711
Laparoscopic	Ref	Ref	Ref
Histopathology			
Complicated or chronic appendicitis	3.793	1.957–7.350	<0.001
Non-complicated acute appendicitis or normal appendix	Ref	Ref	Ref
Age (years)	0.990	0.961–1.020	0.493
BMI (kg/m^2^)	0.974	0.917–1.034	0.389
Temperature (°C)	0.745	0.445–1.245	0.261
Hb (g/dL)	0.854	0.682–1.068	0.166
WBC (K/uL)	1.055	0.994–1.120	0.080
Creatinine (µmol/L)	1.000	0.986–1.014	0.981
Duration of surgery (min)	1.010	1.002–1.018	0.016*

## Discussion

This study aimed to investigate the prevalence of complications after appendectomies and the factors influencing their occurrences. This study found the complication rate after appendectomies to be 10.8%, similar to that reported in previous studies, with multiple studies having reported nearly identical rates [12,13]. Furthermore, although the complication rates were similar between open and laparoscopic appendectomies, SSIs were significantly higher after open than laparoscopic appendectomies. This finding was similar to those of previous studies that found laparoscopic appendectomy to be safe, leading to a quicker return to regular activities and reducing the likelihood of wound complications [9,14-16]. The lack of direct appendiceal contact with the surgical wound during laparoscopic surgery using an endo bag or laparoscopic port, combined with smaller incisions, may result in lower SSI rates with laparoscopic access. In contrast, the rate of intra-abdominal collections was higher after laparoscopic than open appendectomies, exclusively occurring in laparoscopic procedures. These results align with previous research indicating that patients who underwent laparoscopic appendectomies had a higher risk of intra-abdominal abscesses [14,15]. The open approach may reduce the risk of intraperitoneal contamination because the appendix is often divided outside the abdominal cavity, and the stump is inverted following division. In contrast, during laparoscopic appendectomy, the appendix is divided and dissected inside the peritoneal cavity [17]. However, a recent study that included 1809 appendectomies showed that the rates of intra-abdominal abscesses following surgery were nearly similar when comparing open and laparoscopic appendectomies [18]. These results indicate that the risk of intra-abdominal collections may be influenced by several factors other than the type of surgery alone. Katkhouda et al. went further in their study and investigated the effects of the surgeon's laparoscopic skills on the rate of intra-abdominal abscess following appendectomy. Their results showed that the rate of intra-abdominal abscess decreased from 2.4% to 0.4% after they introduced a specialized laparoscopic team for laparoscopic appendectomy [19].

Our study also found that the most common histopathological findings after appendectomy were acute, perforated, and gangrenous appendicitis (72.3%, 19.6%, and 3.4%, respectively). Jat et al. found that acute, suppurative, and gangrenous appendicitis (47.5%, 32.5%, and 11.0 %, respectively) were most commonly reported [20]. In the present study, suppurative and acute appendicitis were counted as one. Therefore, these results were consistent with those reported by Jat et al. Furthermore, the complication rate was higher in the patients with complicated appendicitis. Multiple previous studies reported similar results, as complications occurred more in patients with complicated appendicitis [21,22]. Although histopathology does not change the management plan, it is still required to identify the presence of a carcinoid appendix [23].

Our results also revealed that prolonged operative times significantly influenced the rate of appendectomy complications. A previous study found a relationship between operative times and complications following laparoscopic surgeries [24]. According to Jeon et al., case complexity, rather than direct causation, is the determining factor in the relationship between surgery duration and complication rates. A multivariate analysis accounting for age, sex, BMI, prior surgery, and comorbidities in appendicitis cases demonstrated that extended surgery durations increased the likelihood of complications [25].

In the current study, patients with higher WBC counts and CRP levels presented with a higher risk of complications. Previous studies revealed that the incidence of perforation increases five-fold with an elevated CRP level [26], and a higher CRP level correlates with extended surgery duration [25]. Another study found that increased WBC counts and CRP levels indicated a higher risk of complications before surgery. The risk also increased six-fold when the WBC count was >16.500 K/μL and CRP levels were >3.1 mg/dL [27]. Increased CRP and WBC levels inflict inflammation, which leads to a more difficult operation and higher rates of complications [28].

Furthermore, the median length of stay was three days, similar to that reported in previous studies [29-31]. Patients with complications had significantly more extended hospital stays than those without complications (median: seven days vs. two days). This was supported by a previous study that found that the mean hospital stay in patients with complications (11±7.77 days) was longer compared to that in those without complications (5±2.24 days) [12]. In our study, most patients with complications were classified as Clavien-Dindo grade III (38.3%), which necessitated surgical intervention. This could explain the longer length of stay for those with complications, as further surgical intervention requires more time for scheduling and recovery before discharge.

We also found that one patient had an iatrogenic bladder injury (0.2%) that occurred during a laparoscopic appendectomy. Xavier et al. found, in a recent study, that iatrogenic bladder injuries occurred in two of 1147 cases, with a resampling rate of 0.17% that was consistent with the current study. Additionally, both incidents occurred during laparoscopic appendectomies [32]. One potential explanation for this complication could be the multifaceted nature of our hospital, which serves not only as a tertiary care facility but also as a prominent teaching center. This combination of roles, where advanced and specialized medical care is provided alongside opportunities for medical education and training, may contribute to these complications.

Strengths and limitations

To the best of our knowledge, this study reports the largest sample size for appendectomy outcomes in Saudi Arabia. Additionally, all data were collected from a single center while ensuring a consistent standard of care for all patients. Patients without postoperative notes were excluded to ensure maximum data accuracy. However, this study had the following limitations: (1) some data, such as CRP levels, were missing in some patients, which could have resulted in some inaccuracies, and (2) some patients may have developed complications after discharge but received treatment at a different center, limiting the documentation.

## Conclusions

In conclusion, we found that surgery type did not significantly affect the overall complication rate. However, certain complications occurred more frequently after specific surgery types. Additionally, the histopathology of the appendiceal specimen was significantly related to the occurrence of complications. Moreover, high CRP levels and WBC counts were associated with a higher risk of complications. Although the complication rate of appendectomies was relatively low, the mortality rate was 0%. Postoperative complications should be critically identified to further improve patients’ overall health and quality of life. Factors such as histopathological reports and preoperative laboratory findings may help predict complications following appendectomies. However, considering this study was conducted in a tertiary care center, rates of complications may be lower than those in primary centers. We recommend further research be conducted, including using data from different primary and tertiary centers, to establish more accurate results.
